# Managing Post-Disaster Primary Health Care: Experiences of Public Health Professionals After the 2023 Kahramanmaraş Earthquake

**DOI:** 10.3389/ijph.2025.1608363

**Published:** 2025-11-18

**Authors:** Ceyda Sahan, Ahmet Can Bilgin, Bülent Kılıç, Pelin Bulut, Esra Mert, Seçil Nur Kantaş, Nuri Alp Özünlü, Tuğrul Erbaydar

**Affiliations:** 1 Faculty of Medicine, Dokuz Eylül University, Izmir, Türkiye; 2 Faculty of Medicine, Okan University, Tuzla, Istanbul, Türkiye

**Keywords:** earthquake, disaster management, primary health care, public health professionals, public health services

## Abstract

**Objectives:**

To explore the post-earthquake experiences and perspectives of public health specialists, with a particular focus on the delivery of public health services following the 2023 Kahramanmaraş earthquake.

**Methods:**

This qualitative study employed a phenomenological approach, aiming to capture lived experiences and contextual understanding of the situation. In-depth interviews were conducted with 15 public health specialists. Participants were selected through purposive sampling. Data were collected via in-depth interviews and analyzed thematically using multiple coding by the research team.

**Results:**

Public health specialists reported experiencing stress, burnout, and housing difficulties due to increased workloads and suboptimal working conditions, despite also expressing a sense of professional fulfillment in crisis management. Key challenges included problems in temporary housing, infectious disease control, and insufficient coordination in primary healthcare services and community-based approaches. Poor coordination, vague job roles, and inadequate training and resources were cited as significant barriers to effective disaster response.

**Conclusion:**

The main challenges following the earthquake included increased workloads and unclear role definitions. These findings underscore the importance of establishing pre-defined job descriptions and clear lines of authority to improve disaster preparedness and response in the health sector.

## Introduction

Disaster management in public health encompasses assessing needs, addressing those needs with available resources, preventing secondary health issues resulting from disasters, implementing and evaluating disease control strategies, and enhancing emergency planning for future disasters [1]. In disaster situations, especially in developing countries, key priorities include: situational assessment, immunization, water and sanitation management, food security, provision of shelter, emergency health services, control of infectious diseases and epidemics, public health surveillance, human resources and training, management of transportation and communication operations, resolution of security issues, and ensuring effective coordination [2]. Furthermore, it is imperative to implement preliminary measures [3].

Following the 6.3 magnitude earthquake that struck New Zealand in 2011, the health system faced significant challenges. These included power outages, disruption of communication systems, inadequate pre-hospital care, difficulties in registering and tracking patients, managing agitated patients, coordinating [4]. For instance, in the 2008 Wenchuan earthquake in China, which registered 8.0 on the Richter scale, a comprehensive strategy was implemented to facilitate the response of public health services in the affected regions. An effective and collaborative coordination system for medical rescue forces was established, encompassing areas such as medical rescue force deployment, medical treatment, public health interventions for infectious disease control, and health professional assistance for emergency medical rescue. It was aimed to integrate the system at local and national levels to ensure an effective recovery period. During this phase, the World Health Organization (WHO) provided technical and personnel support to identify risks and analyze demand [5]. Following the 6.5 magnitude earthquake in Bam, Iran, in 2003, a significant number of lives were lost, and issues are concerning water supply, electricity, and telephone connections. Healthcare services were disrupted, and major roads and the airport sustained damage. Under these conditions, there were disruptions in both emergency and preventive health services. In accordance with the national guidelines for natural disasters, the most critical services identified included public health education, environmental health, mental health and personal healthcare services, counseling, first aid, monitoring of drinking and bathing water sources, waste management, and the assessment of fixed and mobile toilets, as well as vaccine storage [6]. Effective public health practices play a crucial role in mitigating damage following earthquakes. Despite the massive scale of the Great East Japan Earthquake, which struck the Sanriku coast of Japan on 11 March 2011, with a magnitude of 9.0 on the Richter scale, no large-scale outbreaks of infectious diseases were reported. This was attributed to the implementation of *ad hoc* surveillance systems, including daily outbreak detection monitoring conducted by public health teams at large-scale assessment centers to monitor the infectious disease situation [7]. This, in addition to emergency rescue and treatment services following an earthquake, providing systematic and adequate food, water, and shelter, especially for vulnerable age groups and individuals with pre-existing conditions, constitutions fundamental public health services [8].

Following the 1999 Marmara earthquake in Turkiye, significant public health issues were highlighted in the tent cities established in the Gölyaka district of Bolu province. Notable problems included inadequate facilities for bathing, laundry, and cooking. Additionally, the communal kitchens in these tent cities often failed to provide meals that met the recommended energy and nutritional requirements [9]. Initially, following the earthquake, the majority of injuries were fatal and included pulmonary, renal injuries, and limb fractures. However, 7 days after the earthquake, a rapid shift in health needs was observed. Respiratory infections, diarrhea, mental health issues, and chronic disease management emerged as primary concerns directly related to public health service delivery [10]. Earthquakes, which are frequently experienced in Turkiye due to geological structure, not only cause public health problems but also require public health experts, who are directly involved in the solution of these problems, to be involved in the process from many perspectives. The magnitude 7.8 Kahramanmaraş-centered earthquake that struck Turkey and Syria on 6 February 2023, caused surface displacements of up to 8 m along the East Anatolian Fault, while a subsequent magnitude 7.6 aftershock generated deeper slips along a separate fault segment [11]. An earthquake of this magnitude caused extensive damage to buildings and infrastructure, leading to substantial casualties and numerous injuries.

Although there are studies in the literature on public health problems experienced in the process in order to meet the needs of the affected people after earthquakes and how this process is managed, studies that include in-depth information on the implementation of public health services after the Kahramanmaraş 2023 earthquake are limited. The aim of this study is to determine the post-earthquake experiences and thoughts of public health specialists who are actively working in the earthquake region, especially in the provision of public health services, after the Kahramanmaraş 2023 earthquake.

## Methods

This study is a qualitative research designed with a phenomenological approach. Public health specialists (15 out of a total of 43 specialists) working in the 5 most affected provinces (Hatay, Kahramanmaraş, Adiyaman, Gaziantep, Malatya) after the February 6 earthquakes have been reached online. In Türkiye, in order to obtain the title of public health specialist, it is mandatory to graduate from public health residency programme after becoming a medical doctor. Public health specialization involves different processes than doctoral education. Public health specialization training includes clinical practical training processes in cardiology, chest diseases, internal diseases, etc. It also includes some topics such as epidemiology, biostatistics, health management, health policies, occupational health, environmental health, communicable diseases, woman health, reproductive health. In addition, there is a requirement to practice in local primary healthcare service provision within the scope of public health specialization [12]. For this reason, public health specialist medical doctors mostly work in primary healthcare services, especially in managerial and technical positions. They are the closest observers of primary healthcare service provision during this disaster, were selected as participants.

Participants were selected through convenience sampling (to be a public health specialist, to be actively working in the most affected earthquake zone during or after the earthquake, and to volunteer to participate in the study). We invited 43 existing specialists and ended up with 15 after data saturation [13, 14]. Two potential participants declined to take part in the study due to time constraints. The 15 participants included public health specialists with varying levels of seniority, years of experience, and institutional affiliations (e.g., provincial health directorates, district health centers, and academia). The researchers and interviewers share the same professional field with the participants as members of the Association of Public Health Specialists, and some participants were already professionally acquainted with certain members of the research team and aware of their academic interest in the study topic. In the study, at least 2 people from each province, 8 of them women and 7 of them men. The average age of the study participants is 34.5. In-depth interviews were carried out 6 months after (between September and August 2023) the earthquake, corresponding to the recovery phase of the disaster management cycle.

The data were collected by conducting in-depth interviews. The interviews were conducted by three public health researchers (BK, CŞ, ACB), all of whom are trained in qualitative research methods and have each published at least three peer-reviewed qualitative research articles indexed in the Web of Science. Our research team consists of eight authors (four female and four male), all working as academic staff at the Department of Public Health in the university. Four of the authors (CŞ, ACB, BK, TE) hold both MD and PhD degrees and have nearly 10 years of experience in qualitative research, with numerous publications in indexed international journals. The others (PB, EM, SNK, NAO) are young medical doctors and public health residents actively engaged in disaster and public health research. A semi-structured interview guide developed by the research team was used, including open-ended questions and follow-up prompts. The experiences of public health specialists related to the earthquake and its aftermath, working conditions, housing conditions, thoughts about the state of public health services, thoughts about national/international institutions, thoughts about specialized education, continuing education and the Turkish Association of Public Health Specialists (HASUDER) were questioned through semi-structured questions. Due to logistical constraints, all interviews were conducted online through secure video conferencing platforms. Online interviews were conducted at participants’ workplaces or homes, scheduled at flexible times during the day to accommodate their availability. All interviews were conducted in privacy, and no individuals other than the participant and the interviewer were present. Informed consent was obtained from all participants for the study, audio recording was made during the interviews and the questions were converted into text. The average interview time is 58 min. Data were analyzed using Braun and Clarke’s thematic analysis framework [15]. Two researchers independently coded the transcripts, discussed discrepancies, and refined the final themes through consensus. The analyses continued as the data were collected and the study was completed with 15 people when the data reached saturation. In order to ensure the validity and reliability of the research, after the interviews were translated into text, “participant confirmation” was sent to the participants. The approval of the ethics committee (Dokuz Eylul University Ethical Committee) was obtained for the research. Detailed information on the study design and data collection process can be found in Supplementary Table 1, which includes the checklist related to this research.

## Results

Participants’ responses fell into five broad themes: Public Health Specialization (Codes: increase in workload, importance of specialized training, experience acquisition, lack of in-service training); Public Health Services (Codes: Temporary Housing Centers, Primary Health Care Services, Surveillance, Community-Oriented Approach); Health Management in Disasters (Codes: Uncertainties-Chaos, Lack of Coordination, Authority Problem); Solidarity (Codes: The Importance of the National Professional Association, The Support of National and International Institutions, Communication) and psychological status (Codes: Anger, Helplessness, Job Satisfaction, Burnout). Table 1 provides illustrative quotations for each theme and codes.

**TABLE 1:** Themes, codes and selected supplementary illustrative quotations (Türkiye, 2023).

Themes	Codes	Illustrative quotations
Public health specialization	Increase in workload	“Of course there was a serious increase in workload, because we worked like that most of the time without the concept of overtime. … As long as you sleep and wake up in the institution, you always feel like you are at work, you do not have a chance to get away because you do not have a place to stay, you are already staying in the institution, so to be honest, there was probably no rest for the first twenty days.” (Participant 12, male, 31 years old) “… I actually …… found food and water here and there, half hungry and half full …, I worked until half past one at night. …… Then I went to the directorate and said, look … put us on shifts, … like everyone else because everyone else was put on shifts by now.” (Participant 15, female, 38 years old) Speaking about the first three months after the earthquake, the workload had increased three to four times. It was like this, professor: I would return home at 1 or 2 a.m., including weekends—we were working non-stop. Because there was work to be done. But let me make a note here: I did all of this voluntarily. No one ever told me, “You must work until 1 or 2 a.m.” or “You must work on weekends.” It was just that the workload was overwhelming, and there were many public health issues to address. (Participant 7, female, 34 years old)
	Importance of specialized training	“When I was an assistant, I also took a disaster and emergency management course at a congress, but some things happen, we are not prepared and there is always something missing. You know, I think there is something missing in the trainings, a little bit in terms of application and internalization…… Not only public health experts, but frankly all managers need to be informed about disaster situations. But something like that is missing right now and continues to be missing.” (Participant 9, female, 44 years old) “As public health specialists, we are the ones with the appropriate training and expertise. However, when everyone tries to have a say, there are people who attempt to tell us how to do our jobs, or even intervene by saying things like 'do not say this' or 'do not do that' right in front of us.” (Participant 13, female, 32 years old.) “I find comfort in the thought that if I was able to contribute, even in a small way, then it was worthwhile. As a public health professional, I would not have wanted to come here three or 5 months later and say, ‘So, what did you do? I hope there was no outbreak and everything went well.’ That would not have felt right to me.” (Participant 7, female, 34 years old)
	Experience acquisition	“But how should I say it, I had the experiences that I could only have seen and lived if I had 30 or 40 years of professional experience, in a period of 6 months. That’s why I am actually very happy, I mean, those were very difficult days, but living this experience firsthand as a public health worker, gaining experience, it was, how should I say it, a great experience in my professional life, I mean, I can say that for my future professional life. In that sense, it was bad but useful” (Participant 7, female, 34 years old) “Yes, we may know things in theory—that’s true—but unless we witness real-life examples, some aspects may not fully settle or make sense, professor. I can say that this was a gap I experienced. Otherwise, I do not think there is any issue with the theoretical training itself.” (Participant 1, male, 34 years old)
	Lack of in-service training	“…You can access information… if you want, with the help of people. But when the mechanisms that make these decisions and make them happen do not work, information alone is not enough.” (Participant 3, female, 44 years old) “There should be various interactive trainings based on different disaster scenarios—and they need to be realistic. They should not be superficial or merely theoretical. We need to truly understand: in that moment, in that situation, what should we do beforehand?” (Participant 7, female, 34 years old.) “I do not think it’s sufficient, because without experiencing it or practicing it somehow, this kind of knowledge does not really align with textbook learning. I believe something more practical is needed—like a readily accessible guide that can be referred to immediately in such situations.” (Participant 8, female, 33 years old.)
Public health services	Temporary housing centers	“… we were going to a place and they were saying there is a tent city here, go and evaluate it, I go, they have already set it up, they have done everything they should not do. From that moment on, there is no point in me saying this will not happen here. Because the manager in front of me is telling me what to do, I cannot change this place from now on. Then it has no point, so I think we are deficient and wrong in that regard.” (Participant 13, female, 32 years old) “Because the buildings are damaged, family physicians cannot work in their own family health centers. So, they rotate—each working a week at a time in the container settlements, while still making sure they follow up with their own registered populations. But since they’re rotating, they do not really develop a sense of ownership over the container sites. And because they do not take that ownership, like I mentioned before, our district health team has to keep following up on those areas continuously.” (Participant 13, female, 32 years old)
	Primary healthcare services	“We have experienced major problems in monitoring and vaccinating pregnant women, babies and children in the last 6 months due to the lack of activities of FHCs and especially because they are not held accountable for their performance.” (Participant 11, male, 31 years old) “In the district where I work, 17 family medicine units have been vacated. Even more nurses have left. So now we’re facing a serious issue: the population is gone, the nurses are gone, and there’s no performance-based payment anymore. Our vaccination rates have dropped significantly, because without support staff—without an assistant nurse—the family physicians cannot carry out antenatal follow-ups or monitor immunizations. They’re only providing outpatient services. We’re still collecting water samples, but they often come back as non-compliant.” (Participant 13, female, 32 years old)
	Surveillance	“I could have taken part in the establishment of communal living spaces, I could have ensured that it was more orderly from the very beginning. I could have kept those records from the very beginning. I could have made a digital version of the Household Identification Forms, and when I placed a recorder there on the first day, I could have fully understood the population in each tent city and provided them with good health services. I was able to intervene after the communal living spaces were established here. We were not invited anyway, but that could have been difficult. We were not effective in the establishment of communal living spaces.” (Participant 14, male, 37 years old.) “I had started collecting some data. Like, ‘How many pharmacies are open?’ or ‘What’s the status of our family health centers?’ I was also curious about the extent of destruction. Sometimes, using an official vehicle, I would drive around more densely populated areas to observe, like, ‘How much damage is there? What’s the situation?’ I’ve been trying to identify problems early on and think about how we can take preventive measures.” (Participant 1, male, 34 years old.)
	Community-oriented approach	“Of course, preventive health services require knowing the region. In other words, you cannot immediately set up a tent and start providing preventive health services. I am aware of this, but their organization can be taken as an example, we can even join, in other words, there can be public health workers in UMKE, this is the first way. I do not know if we can organize our own NGO in this way.” (Participant 14, male, 37 years old) “It should not be verbal or theoretical, or just done for the sake of appearances. I do not think disaster training should take place in hotels or conference halls. Public health professionals need to be out in the field. You cannot train for a disaster by sitting in a meeting room.” (Participant 7, female, 34 years old)
Health management in disasters	Uncertainties-chaos	“So it’s not just the Ministry of Health, but it was chaos from top to bottom. It’s not clear who is responsible for what. It’s a situation where you can either do a lot or nothing. It was a situation like that, to be honest, I can say that it's negative.” (Participant 2, male, 37 years old) “It felt strange—like being in a war movie or one of those end-of-the-world films.”(Participant 13, female, 32 years old)
	Lack of coordination	“Imagine there are three or four governors in a city. Imagine there are two or three provincial health coordinators in a city. Imagine there are four or five public health service directors in a city. So there was chaos.” (Participant 4, male, 33 years old) “If we evaluate the central government’s response—just as an example—there were cases where 6 or 7 sub-governors and one governor were assigned to a single district. In my opinion, that was a mistake. Even if you assign 100 officials, in the end, it was just one person actually running things. So I do not see the point of appointing that many administrators.” (Participant 2, male, 37 years old)
	Authority Problem	Basically, in the district where I work, public health services, in my opinion, the main problem of public health services is that the people who provide public health services have little to do with public health. Our district health director, a general practitioner, may be a great eye-catcher, a good administrator, but in order to organize public health services, you need to listen to the ideas of public health specialists, people who are trained in this field and know the field. (Participant 5, male, 37 years old) “I had made three warnings about the site where the debris was being dumped after the destruction—two related to asbestos, and another about a different issue. At first, I was met with responses like, ‘Noted.’ And for the first couple of days, they seemed to pay attention. But then I saw that it was completely ignored. That was… how can I say… it was a bit bruising, even hurtful.” (Participant 1, male, 34 years old) “By the end of the third month, I had introduced myself to everyone—AFAD, the gendarmerie, the district directorate of national education, the social services department, you name it. And then they started getting back to me. Like, ‘There’s a public health specialist here, let’s coordinate with them.’ As I said before, every institution had set up its own desk and was present in the field in some capacity. The main issue in the early days was a serious lack of coordination.” (Participant 7, female, 34 years old)
Solidarity	The importance of the national professional association	“… My relationship with HASUDER has been since my assistantship, I have been a very close member and also in the working groups, I was quite active before this process. I think it was in the first days of the first earthquake. you called me. I was very emotional like that. At that time, my psychology was really bad and feeling your support, do you need anything? Where are you staying? Can we do something all the time? Even your saying was very good for me. (Participant 6, female, 32 years old) “As HASUDER, I think it would have been better if we had maintained more frequent communication on this issue. But apart from that, I believe we mostly did what needed to be done.” (Participant 1, male, 34 years old) “With HASUDER, I’ve been following their webinars and events closely. There were even some live broadcasts, which were later uploaded to YouTube. For example, I listened to a special broadcast about Hatay afterwards. I think there were two or three reports specifically related to the earthquake as well.” (Participant 7, female, 34 years old)
	The support of national and international institutions	“… it is all about the importance of local government. For me, if I have done a hundred jobs, I have done ninety of them with local government. I think public health should definitely be introduced to local government, to every district governor, to every governor. Or even if they knew what we do, everything would be done better or much more easily if they knew that our job is important in the district. In other words, if I had worked with someone who did not know that we were looking at the general public, I could not have done what I wanted.” (Participant 10, female, 31 years old) ‘… the earthquake happened on Sunday, on Wednesday they forcibly brought down a team from abroad for the first time. … there was really no one in the district. … there was no UMKE, no AFAD. Even they could not reach us. I mean, those who came were already coming to the city center. It took some time for them to disperse to the districts.’ (Participant 10, female, 31 years old) “To be honest, I did not use to have a very positive view of social associations—like foundations or even religious groups and communities. But they actually turned out to be quite helpful. They came and provided food to people for two or 3 months, and I think that was really valuable. The same goes for municipalities—many city municipalities were genuinely helpful and made a positive difference, in my opinion.” (Participant 2, male, 37 years old) “There were civil society organizations involved—they would join in, ask us what we were doing, and then share what they were doing. After that, there would usually be some discussion about what we could do together.” (Participant 13, female, 32 years old)
	Communi-cation	“At that moment you do a quick search or, for example, that HASUDER group was very useful, the WhatsApp group. That group was good. The public health academicians responded from there. For example, for those of us who do not have the opportunity to read literature, they prepared short information notes and sent them to us.” (Participant 10, female, 31 years old) “There was the public health specialists' solidarity group, as you know. We also followed the updates and information shared through WhatsApp or Telegram groups.” (Participant 11, female, 32 years old)
Psychological status	Anger	“Because the state was unprepared, I mean there really was no emergency action plan, I think that’s a crime, after all if you’re running the country you should have an emergency action plan. Otherwise, I think you’re not doing your job properly. I mean, most of the people trapped under the rubble, unfortunately, could not get any help for the first 3–4 days to get them out.” (Participant 2, male, 37 years old) “I think the most important issue is being prepared when the event happens. I mean, okay, let’s put aside earthquake-resistant buildings—that might not be our field directly. But at the very least, there should be a clear plan: what should we do when it happens, which units should be mobilized, which regions should respond if a certain province is affected? Because when you’re prepared, your whole behavior changes.” (Participant 2, male, 37 years old)
	Helplessness	“I saw the collapsed buildings at that moment and I was shocked the moment I saw them. I had a nervous breakdown at that moment, but I tried to pull myself together quickly. … How am I going to keep myself alive? I have to survive here. I have to arrange food, a place to stay, something for myself.” (Participant 11, female, 32 years old) “During the forensic burials, I saw around 60 to 70 bodies over the course of about 3 days. That really took a toll on me emotionally. I mean… what more can I say?” (Participant 1, male, 34 years old.)
	Job satisfaction	“It was a time when I enjoyed working. Because seeing the results of what I did quickly made me very happy. So we did it and the importance of public health, I told the governor that too.” (Participant 10, female, 31 years old) “Professionally, it was also a major learning experience for me as a public health specialist—especially in areas like group management and time management. It really gave me a sense of satisfaction.” (Participant 1, male, 34 years old)
	Burnout	“I had two warnings about the rubble, the place where the rubble was dumped after the collapse. I had two warnings about asbestos and one about the other, a total of three. So, to see that they paid attention to it for the first 1–2 days and then did not really pay attention to it was a bit like that, how should I say it, a bit hurtful, a bit like that.” (Participant 1, male, 34 years old) I called the District Governor twice, crying. … I said, “I will resign.” You know, I’m crying on the one hand. On the other hand, I’m doing something like, “They’re pushing me too hard, I cannot do it.” Of course, there were some days when I had a hard time. Thank God, he was always there for me, I cannot do him any favors on that. (Participant 10, female, 31 years old) “Then, like I said, my motivation dropped—and that’s when a kind of indifference started to set in. It was a bit dangerous, actually, because I had reached a point where I barely cared about anything. Not just in a professional sense, but also in my personal life.” (Participant 1, male, 34 years old)

### Theme 1: Public Health Specialization

It has been found that public health specialists are in a state of stress, anxiety, fear, sadness, helplessness, frustration and burnout with increasing workload, fatigue and difficulty of working conditions, as well as housing problems during this period. Besides this, it was emphasized that the specialized training of public health specialists is theoretically sufficient, but there are different aspects of field applications in disaster situations. It was also found that they drew attention to the lack of in-service training on disaster issues from the point of view of public health specialists. It has also been found that public health specialists achieve spiritual satisfaction due to using their professional experience in crisis and disaster management with a cold-blooded approach. However, it has also been frequently stated that despite the increase in the workloads of public health specialists, insufficient authority is not given in the field and performance-based pay problems lead to loss of personal rights.

The primary tasks of public health specialists have been identified as determining priorities in the field, organization, coordination, surveillance, data collection and contact tracing. Experts highlighted the positive aspects of cooperation, teamwork and communication, as well as individual efforts of public health specialists at each stage. They stated that working on the crisis desk, taking on additional responsibilities, planning and organizing have provided them with important professional experience.

### Theme 2: Public Health Care Services

In terms of public healthcare services, it was stated that there was not enough cooperation with public health specialists, especially when establishing temporary housing centers, and therefore there are problems such as infrastructure, flood and fire risk in temporary housing centers. The most common public health problems in the field of communicable diseases, there is a lack of immunization, hygiene problems, scabies, measles and acute gastroenteritis. In the field of non-communicable diseases, there is a problem of chronic patient follow-up and medication. In the field of environmental health, there are dust and asbestos, water, sewage, waste management problems. Reproductive health services are usually not provided. While the provision of mobile services is found necessary and positive, migrant health, mental health, elderly health, occupational health and Cancer Early Diagnosis, Screening and Education Center (KETEM) services generally contain deficiencies. It has been found that there are serious problems in primary healthcare services and surveillance. The most important problems were identified as the shortcomings in the community-oriented approach and regular data collection and analysis in primary healthcare. In addition, in terms of primary healthcare services, Family Health Centers (FHCs); it has been reported that FHCs are damaged, FHCs’ employees have left the city, the remaining staff work alternately, staff shortages and service planning problems have disrupted due to. It has been stated that failure to reduce the performance practice in FHCs has good and bad consequences for health services.

### Theme 3: Health Management in Disasters

Problems related to inadequacies in the appointment of public health specialists as managers, uncertainties in job descriptions, lack of specialists and coordination problems, technical and infrastructure deficiencies have been identified in the field of health management. In addition, managerial problems such as lack of in-service training, inadequacies in managing permits, low motivation, improper distribution of facilities and forcing health workers to work in earthquake zones were also mentioned. In addition, administrative problems such as uncertainties, the formation of a chaotic environment, and the problem of authority have come to the fore.

### Theme 4: Solidarity

It has been seen that the activities of the Turkish Association of Public Health Specialists (HASUDER) have positively affected solidarity and sense of belonging during this period, and academic support and consulting activities on disaster management have been received quite positively. It has been found that communication groups such as whatsapp and telegram have a positive impact on communication in the post-earthquake period. Experts have stated that the effectiveness of HASUDER is gradually increasing and HASUDER publications are very useful. They stated that a sense of belonging is felt thanks to the association and that membership in the specialty association is necessary. The interviewed experts reported both positive and negative opinions about some national organizations; Disaster And Emergency Management Presidency (AFAD), Turkish Red Crescent (Kızılay), National Medical Rescue Team(UMKE), ministries, municipalities, non-governmental organizations (NGOs), in addition to the fact that cooperation with national and international institutions is positive in terms of solidarity. On the other hand, it has been seen that international organizations are more experienced and make a positive contribution to the process in terms of supervision and service provision.

### Theme 5: Psychological Status

In the post-earthquake period, it has been observed that public health specialists experience feelings such as anger and helplessness in the face of administrative problems and the destruction caused by the earthquake in the first place. In addition, they experienced a more satisfactory period in terms of job satisfaction, but they were still challenged and experienced burnout due to the increase in workload and the lack of equipment related to the disaster.

**FIGURE 1:**
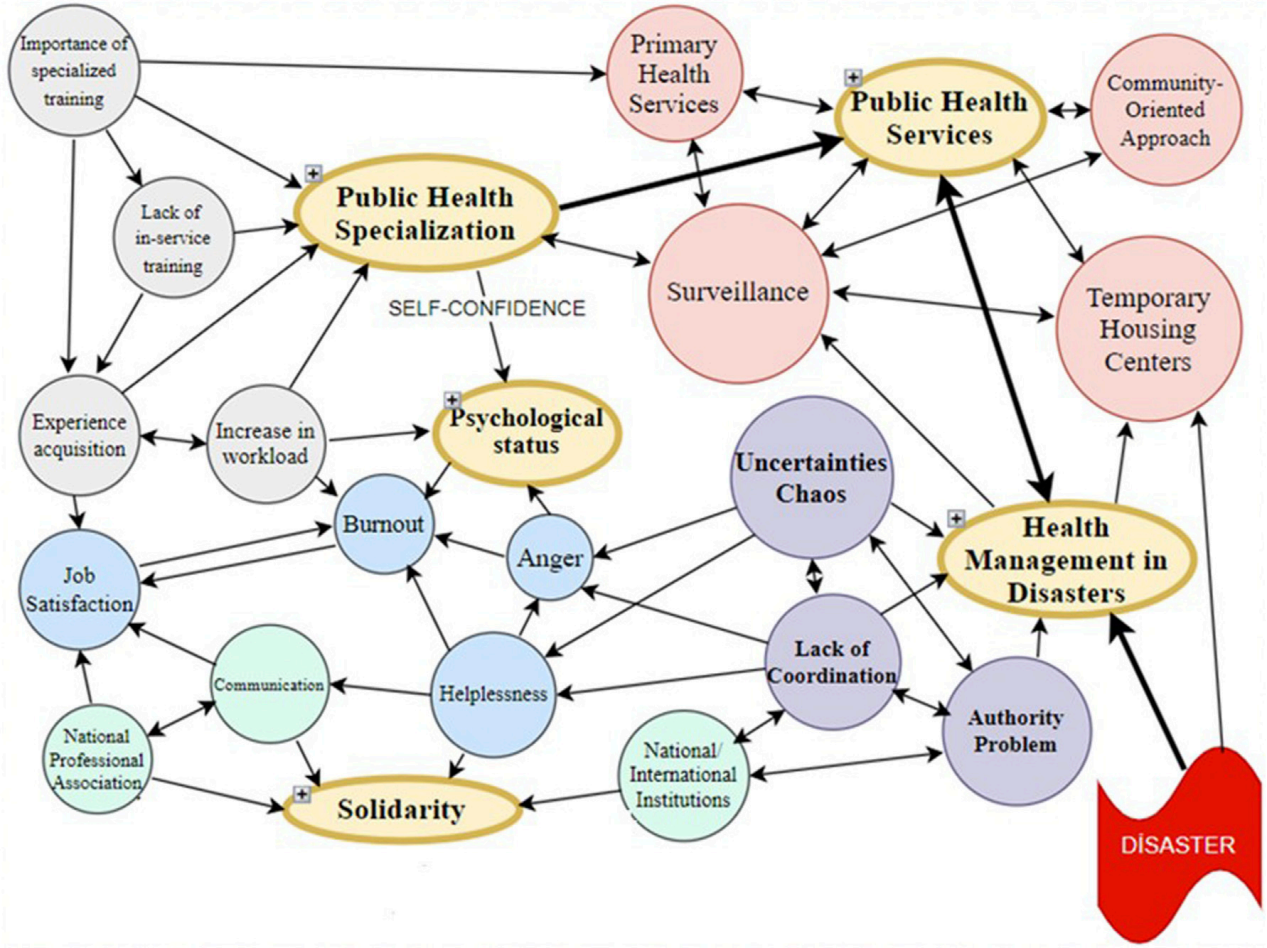
The coding tree (Türkiye, 2023).

The coding tree in Figure 1 reflects a multidimensional and interrelated structure of public health professionals’ experiences in the aftermath of the 2023 Kahramanmaraş earthquake. At the center lies the theme of Public Health Specialization, which forms the foundation for understanding professionals’ roles, responsibilities, and preparedness. This foundational theme is closely linked to Public Health Services, where practical challenges—such as disruptions in service delivery, surveillance gaps, and weakened community-based approaches—manifest in the field. These challenges are exacerbated by deficiencies in Health Management in Disasters, particularly the lack of coordination, unclear authority structures, and administrative bottlenecks. The theme of Solidarity emerges as both a mitigating and reinforcing factor: while weak institutional coordination creates tension, professional networks (e.g., HASUDER) and inter-organizational collaboration provide emotional and operational support. Lastly, the theme of Psychological Status both influences and is influenced by the other domains; emotional responses such as burnout, helplessness, and fulfillment arise as consequences of structural, managerial, and interpersonal dynamics. The interconnections among these themes demonstrate that effective disaster response in public health requires not only technical capacity but also coordinated leadership, professional solidarity, and psychological resilience.

## Discussion

This study aimed to explore the experiences of public health professionals in primary healthcare and disaster management in Türkiye following the 2023 Kahramanmaraş earthquake, with the goal of drawing lessons to inform the improved management of public health services.

One of the most significant issues identified was the psychological trauma experienced by public health professionals in the aftermath of the disaster. Increased workloads and housing difficulties during this period created challenging working conditions. However, their professional expertise and familiarity with the region enhanced their capacity to play a key role in delivering public health services, which also contributed to a sense of professional satisfaction and growth. Field priorities included organization, coordination, surveillance, the establishment of temporary housing centers, infectious disease control, environmental health, and maternal and child health services. Nevertheless, various shortcomings were observed: damage to infrastructure, loss of life among health personnel or their relatives, shortages of medical supplies, unclear job descriptions, and weak leadership in field-level health service management. A noteworthy positive aspect was the support provided by national and international non-governmental organizations, which offered both material and moral contributions through their infrastructure and workforce. However, this process underscored the need for national disaster management systems capable of effectively coordinating the roles of supporting NGOs.

Findings indicate that a more systematic and strategic approach is needed in delivering public health services—both in re-prioritizing during disasters and managing the workforce. It is particularly important for preventive health service providers to be well-acquainted with the affected region and its population. To this end, public health professionals and medical personnel should be dynamically oriented through in-service training before being deployed. While it may be advantageous for staff to have previously worked in the region, it is not always sustainable to rely on individuals who have personally experienced the disaster. As reported in this study, the majority of interviewed public health professionals experienced stress, anxiety, fear, sadness, helplessness, frustration, and burnout. These findings align with existing literature indicating the mental health risks faced by healthcare workers in disaster contexts [16–18]. Those suffering from burnout, post-traumatic stress disorder, anxiety, or depression should, whenever possible, be removed from the field and provided with appropriate care [19]. In order to protect health workers from many occupational hazards during crisis periods, it is also necessary for these employees to acquire skills learned through structured education and training with personal characteristics such as adaptation and resilience before a disaster [20]. It has been seen that psychoeducation initiatives, especially after the disaster, have an important effectiveness in understanding and coping with stress. In addition, it has been observed that teamwork and social support between employees increase the motivation of health workers during this period [21]. Disaster medical relief teams in Canada emphasized the importance of self-care and survival skills, flexibility, adaptation, innovation and improvisation abilities in a study that investigated the effects of non-clinical basic competencies and professional teamwork after a disaster [22].

In this study, it was also emphasized that there are problems related to health service management, especially inadequacies in the appointment of public health specialists, uncertainties in job descriptions and coordination problems, technical and manpower infrastructure deficiencies. Accordingly, it is emphasized that policymakers will make an important contribution to determining the roles of public health specialists, especially at the regional and national levels in emergency and disaster management. Similarly, it is known that the necessary infrastructure should be provided so that search and rescue teams can contribute to the planning of disaster prevention and response strategies in order to improve disaster management. Search and rescue teams also stated that the most important problem in the provision of services in the region is the lack of coordination and provision of water, food and housing conditions for the work of the teams. In addition, it has been experienced that volunteer citizens who come for search and rescue can cause more harm than good due to lack of education and not knowing what to do [23].

After the Kahramanmaraş earthquake, 15 million people were affected by the earthquake in 11 cities in this region of Turkey [24], many hospitals have been partially or severely damaged, and all health services have been disrupted due to this. Especially in the first stage after the earthquake, the medical staff tried to cope with the fatal injuries and health problems among all this Decadence [25, 26]. In particular, disaster victims were found to be more likely to suffer from hypothermia and other health problems due to logistical challenges [27]. Especially in the early days, it was not possible for medical personnel to provide services even in hospitals that were available. Many medical personnel or their relatives lost their lives in the earthquake. AFAD, Ministry of Health, Ministry of National Defense and Minister of Agriculture and Forestry provided health services by ship, aircraft carrier and helicopter and more than 50 thousand patients were transferred to different cities. With the implementation of these efforts, it was observed that the technical efficiency of health services provided in the earthquake-affected provinces increased from an average of 52% on the 5th day of the earthquake to 80% on the 10th day [28].

The first 24–72 h after the earthquake are critical for search and rescue teams, while the next first 2 weeks are important for emergency shelter and relief activities [24, 29]. It is clear that in addition to the provision of emergency clinical services in subsequent periods, the provision of public health services after disasters is also very important. During this season, the fact that the weather has cooled down to −10° at night in some regions and there are also snowy regions has had a very negative impact on the living conditions of the homeless in particular. Due to these cold conditions after the earthquake, collective living in closed areas such as solid football stadiums, schools, etc. has increased and these conditions have brought with them an increased risk of infectious diseases [25]. In terms of emergency shelter, although many earthquake victims were evacuated to different cities, the number of tents and campgrounds required at the first stage for those who remained was insufficient. Some tents have been submerged in the rains due to the unsuitability of the areas where they were set up. Fire incidents have also been observed in tents. Many tents were not suitable for winter conditions, so adequate heating has not been provided [24]. In addition, electricity, telephone, internet, natural gas and water supply infrastructure have been damaged in many cities [25].

After a devastating earthquake in Sichuan province of China in 2008, priority problems in public health services were identified as interruption of access to safe water and sanitation, population displacement, overcrowding, increased risk of infectious diseases and access to health services as priority problems. Within 10 days after the earthquake, 116,700 public health professionals were assigned to work in the region; the process was carried out in coordination with the relevant teams and partner provinces. As a result of the systematic approach taken in disease prevention and control during this period, no disease outbreaks were reported in any of the affected regions, and after the first weeks, public health intervention shifted from disease control and prevention to the restoration of local medical and public health systems and the establishment of local infectious control efforts. Although it was an exemplary approach in this respect, there were also criticisms regarding the lack of a national disaster management institution at that time, the lack of coordination between state institutions at national and local levels, the problem of communication between sectors, the lack of relevant training for intervention in the context of earthquakes, the inadequacy of existing emergency plans, the lack of clarity of relevant state institutions regarding their own functions and responsibilities, and the failure to collect and analyze information in a timely manner. In addition, it was stated that there was no adequate management for the control and distribution of donations, and therefore there was waste and inappropriate distribution [30].

In the Kobe earthquake in Japan in 1995, although public health centers and local health departments were expected to coordinate health-related activities in each region, their efficiency and coordination were highlighted as being quite low due to the lack of clear instructions based on appropriate emergency guidelines for such a major disaster. It has been stated that it is an expected situation that coordinated work will be quite difficult due to reasons such as the dysfunction of important local organizations in previous earthquakes in the country, the different working systems of different aid teams, and the existence of inefficient individualism among some aid workers. It was emphasized that in order to implement effective relief coordination, emergency coordination structures should be clarified in disaster plans at all levels [31].

With climate change, it is expected that there will be many disasters and societies affected by them in the next century. Therefore, it is necessary to reduce disaster risk at national and international levels, prepare applicable emergency plans and provide cross-country support in emergency disaster response. In all these processes, sustainable preparations should be made for preventive health services as well as emergency health service planning [32]. There is substantial evidence indicating that systematically designed models, jointly organized by academic and non-academic institutions, yield more effective outcomes in enhancing public health emergency preparedness and community resilience [33]. This study provides evidence for the importance of predefined roles and coordinated efforts among multiple institutions in the delivery of public health services during emergencies.

Findings from another qualitative study conducted with first responders during the Kahramanmaraş earthquake also highlight challenges in inter-agency coordination [34]. Disasters require coordinated action across multiple sectors. Effective collaboration among first responders, local authorities, national institutions, NGOs, and international actors enables more comprehensive and efficient disaster management by combining diverse resources and expertise [35, 36].

Based on the findings of this study, a proactive and integrated model is proposed to strengthen the role of public health specialists in disaster management. The model emphasizes pre-disaster preparedness, in-disaster coordination, and post-disaster recovery, all of which require structured roles, inter-sectoral communication, and capacity building. Table 2 presents the structure and components of the proposed model for improved disaster management, based on the experiences of public health professionals.

**TABLE 2:** Proposed model: Enhancing the role of public health specialists in disaster (Türkiye, 2023).

Explanation of model components
1. Pre-disaster Preparedness: - Define clear job descriptions and authority levels for public health specialists - Conduct region and disaster specific in-service training and scenario-based simulations - Establish public health focused and integrated plans with national disaster system - Build community trust and local knowledge through ongoing outreach and surveillance
2. Disaster response Coordination: - Assign public health specialists to disaster management system with decision-making roles - Ensure real-time data flow, active surveillance and adaptive response planning - Facilitate cooperation between health, municipality, governor and NGO sectors - Deploy mobile teams focused on hygiene, immunization, community mental health, reproductive, sexual health and surveillance
3. Post-disaster recovery & Evaluation: - Monitor long-term health indicators (community mental health, NCDs, environmental risks, rehabilitation) - Engage in policy feedback loops based on field data - Support the resilience and wellbeing of health workers through psychosocial programs - Document lessons learned to update training and disaster plans

### Strengths and Limitations

This study captures firsthand, valuable, timely and rich qualitative data from frontline public health specialists directly involved in disaster response. It highlights operational and policy-level issues in post-disaster public healthcare delivery. The use of thematic analysis and multiple coders strengthens the validity and rigor of the findings. Reflections on mental health and burnout add depth to operational insights. Although only five cities were selected—those most severely affected by the earthquake—we were able to reach nearly half of the public health specialists working in the impacted region. Given that HASUDER holds no conflicts of interest and maintains no external affiliations with field personnel, the study was conducted under conditions conducive to impartial observation. While the emphasis on the roles and challenges faced by public health specialists may be perceived as a potential source of bias, this focus is consistent with the study’s primary aim and is therefore considered methodologically appropriate. As can be seen from previous studies, the close collaboration of research teams with local teams during these processes facilitates the acquisition of accurate information and creates an environment of trust [37].

Main limitation was lack of triangulation due to earthquake conditions. Data collected from only public health specialists by in-depth interviews. Convenience sampling and voluntary participation may introduce selection bias. Some level of subjectivity may remain, as the study relies solely on the perspectives of public health specialists. That’s why the results cannot be generalized to the other professional (e.g., nurses, other clinicians) or community (e.g., patients, administrators) perspectives. The absence of a comparative perspective (e.g., pre-disaster conditions or other regions) restricts the scope of analysis. The study context is specific to Turkiye and the particular 2023 earthquake, which may limit transferability to other settings or disaster types.

### Conclusion

This research has shown that the theoretical training received by public health specialists on disasters is sufficient. In addition, it contains important findings regarding the post-disaster operational chaos, the increase in workload, the problem of authority and inadequate management. Disaster management is particularly difficult when public health specialists have unclear assignments and role definitions within the health system during disasters. In every province, the job descriptions and authorities of public health specialists, both in crisis management during disaster periods and before the disaster, should be planned in advance and appropriate arrangements should be made accordingly. In addition, the fact that solidarity comes to the fore in earthquakes and similar situations shows the importance of communication channels and specialized associations. These structures should be protected and further developed. It should not be forgotten that healthcare professionals may experience feelings such as anger, helplessness, and burnout in disaster situations such as earthquakes.

## Data Availability

Dataset is not able to be deposited due to being a qualitative study. But the authors can share more information if someone is requested for metasynthesis or other studies.

## Abbreviations

WHO, World Health Organization; HASUDER, The Turkish Association of Public Health Specialists; KETEM, Cancer Early Diagnosis, Screening and Education Center; FHCs, Family Health Centers; AFAD, Disaster And Emergency Management Presidency; Kızılay, Turkish Red Crescent; UMKE, National Medical Rescue Team; NGOs, Non-governmental organizations.
