# Supplementary material for: Managing Post-Disaster Primary Health Care: Experiences of Public Health Professionals After the 2023 Kahramanmaraş Earthquake
Source: Int J Public Health. 2025 Nov 18;70:1608363. doi: 10.3389/ijph.2025.1608363 (PMC12669067; doi:10.3389/ijph.2025.1608363)
Supplement: Supplementary file 1 [file Table1.docx]

Supplementary File 1: COREQ checklist ..

Consolidated criteria for reporting qualitative studies (COREQ): 32-item checklist

Developed from:

Tong A, Sainsbury P, Craig J. Consolidated criteria for reporting qualitative research (COREQ): a 32-item checklist for interviews and focus groups. International Journal for Quality in Health Care. 2007. Volume 19, Number 6: pp. 349 – 357

| **Item No** | | **Guide Questions/Description** | **Section** |  |  |
| --- | --- | --- | --- | --- | --- |
| **Domain 1: Research team and reflexivity** | | | |  |  |
| **Personal Characteristics** | | | |  |  |
| 1. Interviewer/ facilitator | | Which author/s conducted the interview or focus group? | Methods | Three of the eight authors (CŞ, ACB, BK) conducted interviews |  |
| 2. Credentials | | What were the researcher’s credentials? E.g., PhD, MD | Methods | Four of the authors MD, PhD (CŞ, ACB, BK, TE) and others are MD and public health residents (PB, EM, SNK, NAO) |  |
| 3. Occupation | | What was their occupation at the time of the study? | Methods | They have been working at the Department of Public Health as academic staff in University. |  |
| 4. Gender | | Was the researcher male or female? | Methods | Four male and four female |  |
| 5. Experience and training | | What experience or training did the researcher have? | Methods | Four of the eight authors (CŞ, ACB, BK, TE) have experience in qualitative research area nearly for 10 years. They published many qualitative papers in the indexed international journals. The others are young researchers. |  |
| **Relationship with participants** | | | |  |  |
| 6. Relationship established | | Was a relationship established prior to study commencement? | Methods | Researchers and interviewers are in the same public health arena as common members of the Association of Public Health Specialist |  |
| 7. Participant knowledge of the interviewer | | What did the participants know about the researcher? e.g. personal goals, reasons for doing the research? | Methods | The participants know some of the researchers as professionally and their goals for doing the research. |  |
| 8. Interviewer characteristics | | What characteristics were reported about the interviewer/facilitator? e.g. Bias, assumptions, reasons and interests in the research topic | - | No |  |
| **Domain 2: study design** | | |  |  |  |
| **Theoretical framework** | | |  |  |  |
| 9. Methodological orientation and Theory | What methodological orientation was stated to underpin the study? e.g. grounded theory, discourse analysis, ethnography, phenomenology, content analysis | Methods | Phenomenological design (sharing experiences about earthquake disaster) |  |  |
| **Participant selection** | | |  |  |  |
| 10. Sampling | How were participants selected? e.g., purposive, convenience, consecutive, snowball | Methods | Participants were selected through purposive sampling (to be a public health specialist, to be actively working in the most affected earthquake zone during or after the earthquake, and to volunteer to participate in the study). In the study, at least 2 people from each province, 8 of them women and 7 of them men, 15 public health specialists were interviewed. |  |  |
| 11. Method of approach | How were participants approached? e.g., face-to-face, telephone, mail, email | Methods | On-line |  |  |
| 12. Sample size | How many participants were in the study? | Methods | There were 43 public health specialists in the 5 most affected provinces. We invited 43 existing specialists and ended up with 15 after saturation. |  |  |
| 13. Non-participation Setting | How many people refused to participate or dropped out? Reasons? | Methods | Two potential participants declined to take part in the study due to time constraints. |  |  |
| 14. Setting of data collection | Where was the data collected? e.g., home, clinic, workplace | Methods | Online interviews were conducted at participants’ workplaces or homes, scheduled at flexible times during the day to accommodate their availability. |  |  |
| 15. Presence of nonparticipants | Was anyone else present besides the participants and researchers? | Methods | No |  |  |
| 16. Description of sample | What are the important characteristics of the sample? e.g. demographic data, date | Methods | In the study, at least 2 people from each province, 8 of them women and 7 of them men, 15 public health specialists were interviewed. The average age of the specialists is 34.5. The interviews were held between September and August 2023. |  |  |
| **Data collection** | | |  |  | No |
| 17. Interview guide | Were questions, prompts, and guides provided by the authors? Was it pilot tested? | Methods | Yes, a semi-structured interview guide developed by the research team was used, including open-ended questions and follow-up prompts. |  |  |
| 18. Repeat interviews | Were repeat interviews carried out? If yes, how many? | - | No |  |  |
| 19. Audio/visual recording | Did the research use audio or visual recording to collect the data? | Methods | Yes |  |  |
| 20. Field notes | Were field notes made during and/or after the interview or focus group? | - | No |  |  |
| 21. Duration | What was the duration of the interviews or focus group? | Methods | The average interview time is 58 minutes. |  |  |
| 22. Data saturation | Was data saturation discussed? | Methods | Yes |  |  |
| 23. Transcripts returned | Were transcripts returned to participants for comment and/or correction? | Methods | Yes, transcripts were returned to participants for validation (member checking), and their approval was obtained before analysis. |  |  |
| **Domain 3: analysis and findings** | | |  |  |  |
| **Data analysis** | | |  |  |  |
| 24. Number of data coders | How many data coders coded the data? | Methods | Each interview was coded by at least 2 researchers and totally 7 of eight researchers coded the data |  |  |
| 25. Description of the coding tree | Did the authors provide a description of the coding tree? | Results | Yes |  |  |
| 26. Derivation of themes | Were themes identified in advance or derived from the data? | Results | While no fixed themes were determined beforehand, the interview framework was shaped by concepts drawn from the literature and researchers’ field observations. The actual themes emerged inductively during the analysis of interview data. |  |  |
| 27. Software | What software, if applicable, was used to manage the data? | - | No. The data were coded manually and analyzed by thematic content analysis method. |  |  |
| 28. Participant checking | Did participants provide feedback on the findings? | - | No |  |  |
| **Reporting** | | |  |  |  |
| 29. Quotations presented | Were participant quotations presented to illustrate the themes/findings? Was each quotation identified? e.g., participant number | Table 1 | Yes |  |  |
| 30. Data and findings consistent | Was there consistency between the data presented and the findings? | Table 1 | Yes. There was a clear alignment between the data and the findings, which were grounded in participants’ narratives and supported by illustrative quotations. |  |  |
| 31. Clarity of major themes | Were major themes clearly presented in the findings? | Results | Yes |  |  |
| 32. Clarity of minor themes | Is there a description of diverse cases or a discussion of minor themes? | - | No |  |  |
